# A Machine Learning Model for Identifying Sexual Health Influencers to Promote the Secondary Distribution of HIV Self-Testing Among Gay, Bisexual, and Other Men Who Have Sex With Men in China: Quasi-Experimental Study

**DOI:** 10.2196/50656

**Published:** 2024-04-24

**Authors:** Yuxin Ni, Ying Lu, Fengshi Jing, Qianyun Wang, Yewei Xie, Xi He, Dan Wu, Rayner Kay Jin Tan, Joseph D Tucker, Xumeng Yan, Jason J Ong, Qingpeng Zhang, Hongbo Jiang, Wencan Dai, Liqun Huang, Wenhua Mei, Yi Zhou, Weiming Tang

**Affiliations:** 1 Dermatology Hospital of Southern Medical University Guangzhou China; 2 University of North Carolina at Chapel Hill Project-China Guangzhou China; 3 Department of Health Law, Policy, and Management Boston University School of Public Health Boston, MA United States; 4 Institute for Healthcare Artificial Intelligence Application Guangdong Second Provincial General Hospital Guangzhou China; 5 Faculty of Data Science City University of Macau Macao SAR China; 6 Department of Social Welfare University of California Los Angeles, CA United States; 7 Health Service and System Research Programme Duke-NUS Medical School Singapore Singapore; 8 Zhuhai Xutong Voluntary Services Center Zhuhai China; 9 Department of Social Medicine and Health Education, School of Public Health Nanjing Medical University Nanjing China; 10 Saw Swee Hock School of Public Health National University of Singapore and National University Health System Singapore Singapore; 11 Department of Clinical Research, Faculty of Infectious and Tropical Diseases London School of Hygiene and Tropical Medicine London United Kingdom; 12 University of North Carolina at Chapel Hill Chapel Hill, NC United States; 13 Department of Community Health Sciences, Fielding School of Public Health University of California Los Angeles, CA United States; 14 Central Clinical School Monash University Melbourne Australia; 15 Musketeers Foundation Institute of Data Science The University of Hong Kong Hong Kong China (Hong Kong); 16 Department of Pharmacology and Pharmacy, LKS Faculty of Medicine The University of Hong Kong Hong Kong China (Hong Kong); 17 Department of Epidemiology and Biostatistics, School of Public Health Guangdong Pharmaceutical University Guangzhou China; 18 Zhuhai Center for Disease Control and Prevention Zhuhai China

**Keywords:** artificial intelligence, HIV testing, key opinion leaders, machine learning, men who have sex with men, self-testing

## Abstract

**Background:**

Sexual health influencers (SHIs) are individuals actively sharing sexual health information with their peers, and they play an important role in promoting HIV care services, including the secondary distribution of HIV self-testing (SD-HIVST). Previous studies used a 6-item empirical leadership scale to identify SHIs. However, this approach may be biased as it does not consider individuals’ social networks.

**Objective:**

This study used a quasi-experimental study design to evaluate how well a newly developed machine learning (ML) model identifies SHIs in promoting SD-HIVST compared to SHIs identified by a scale whose validity had been tested before.

**Methods:**

We recruited participants from BlueD, the largest social networking app for gay men in China. Based on their responses to the baseline survey, the ML model and scale were used to identify SHIs, respectively. This study consisted of 2 rounds, differing in the upper limit of the number of HIVST kits and peer-referral links that SHIs could order and distribute (first round ≤5 and second round ≤10). Consented SHIs could order multiple HIV self-testing (HIVST) kits and generate personalized peer-referral links through a web-based platform managed by a partnered gay-friendly community-based organization. SHIs were encouraged to share additional kits and peer-referral links with their social contacts (defined as “alters”). SHIs would receive US $3 incentives when their corresponding alters uploaded valid photographic testing results to the same platform. Our primary outcomes included (1) the number of alters who conducted HIVST in each group and (2) the number of newly tested alters who conducted HIVST in each. We used negative binomial regression to examine group differences during the first round (February-June 2021), the second round (June-November 2021), and the combined first and second rounds, respectively.

**Results:**

In January 2021, a total of 1828 men who have sex with men (MSM) completed the survey. Overall, 393 SHIs (scale=195 and ML model=198) agreed to participate in SD-HIVST. Among them, 229 SHIs (scale=116 and ML model=113) ordered HIVST on the web. Compared with the scale group, SHIs in the ML model group motivated more alters to conduct HIVST (mean difference [MD] 0.88, 95% CI 0.02-2.22; adjusted incidence risk ratio [aIRR] 1.77, 95% CI 1.07-2.95) when we combined the first and second rounds. Although the mean number of newly tested alters was slightly higher in the ML model group than in the scale group, the group difference was insignificant (MD 0.35, 95% CI –0.17 to –0.99; aIRR 1.49, 95% CI 0.74-3.02).

**Conclusions:**

Among Chinese MSM, SHIs identified by the ML model can motivate more individuals to conduct HIVST than those identified by the scale. Future research can focus on how to adapt the ML model to encourage newly tested individuals to conduct HIVST.

**Trial Registration:**

Chinese Clinical Trials Registry ChiCTR2000039632; https://www.chictr.org.cn/showprojEN.html?proj=63068

**International Registered Report Identifier (IRRID):**

RR2-10.1186/s12889-021-11817-2

## Introduction

Globally, gay, bisexual, and other men who have sex with men (GBMSM) exhibit a significantly higher median HIV prevalence of 7.5%, in contrast to the global median of 0.7% [1]. In China, GBMSM are also disproportionately affected by HIV, accounting for 25.5% of new HIV infections in 2018 (vs 6.1% in 2008) [2]. Despite the efforts in HIV control, the HIV testing coverage among Chinese GBMSM is still low. In 2019, the national HIV prevalence of GBMSM was 6.3%, yet only 56% knew their HIV status in China [3]. Enhancing people’s awareness of their own HIV status could accelerate the initiation of treatment. People living with HIV who start treatment earlier have a higher likelihood of improving their survival and can also prevent HIV transmission by maintaining an undetectable viral load status (undetectable=untransmittable) [4].

Preventing HIV by increasing HIV testing rates is a key pillar in reaching the first “95-95-95” target, proposed by UNAIDS (the Joint United Nations Programme on HIV/AIDS), that 95% of all individuals living with HIV will be aware of their HIV status by 2025 [5]. One of the innovative approaches to expanding HIV testing coverage is HIV self-testing (HIVST). Specifically, HIVST is an effective decentralized testing tool that allows persons to test in a comfortable and private setting and mitigates concerns about inadvertent disclosure and confidentiality protection associated with facility-based testing [6]. In addition, HIVST may present an opportunity to address barriers that GBMSM may encounter when seeking care in health care facilities [7]. These barriers include but are not limited to, lack of access to health care, fear of HIV-related stigma, perceived discrimination, lack of confidence in health services, and limited knowledge and awareness about HIV [6-9]. HIVST can be conducted with or without assistance in various settings to reach diverse populations [10,11]. Directly assisted HIVST involves in-person guidance from trained providers or peers, while unassisted HIVST relies solely on manufacturer instructions [12]. A systematic review and meta-analysis indicated that self-testers achieve HIVST results with reliability and accuracy similar to trained health care workers [13]. In light of this, secondary distribution of HIV self-testing (SD-HIVST) was conducted within the unassisted HIVST implementation strategy.

In 2023, the World Health Organization (WHO) recommends expanding the use of HIVST services; one of the recommendations is using individuals’ sexual and social networks to promote HIVST and increase testing coverage [14]. One promising model is the social network–based SD-HIVST. As an innovative social network method, SD-HIVST is promising to help reach naïve testers and increase HIVST uptake among men who have never tested before [15-17]. The strategy of SD-HIVST involves individuals (defined as “index”) obtaining multiple HIVST kits and distributing them face-to-face or through peer referral (PR) links on the web to members within their social networks (defined as “alters”) [15,16]. There is increasing evidence demonstrating the effectiveness of SD-HIVST in improving HIV testing coverage and case identification worldwide [15-20]. In China, Wu et al [15] adapted the SD-HIVST model into a social media–based existing HIVST service provided by a GBMSM-friendly community-led organization. Wu et al [15] also included components to improve test accuracy and result return rates. Building on the progress of Wu et al [15], Zhou et al [16] conducted a randomized trial with a modified model by providing monetary incentives to GBMSM index participants who motivated alters to upload their results to the web-based system. The study concluded that SD-HIVST with economic incentives could effectively promote SD-HIVST and encourage newly tested receivers to test. Consequently, this adapted digital secondary distribution strategy was culturally appropriate in the Chinese setting, where GBMSM influencers hold a premise in HIVST distribution to their social contacts with limited access to self-testing [15,16].

Sexual health influencers (SHIs) are individuals with a strong influence on disseminating information about HIV and sexually transmitted infections (STIs) within their social networks [21-23]. Studies have shown that those at the center of their networks may influence their peers’ adoption of healthier behaviors [21,22]. It was also reported that SHIs are important for improving HIV care-related services. For example, a study has shown that GBMSM with strong influence tend to participate in more sexual health campaigns and use HIV and syphilis testing [21]. The social comparison theory states that SHIs might serve as role models for well-being and self-improvement within the GBMSM community, offering an ideal to which other GBMSM can strive [24].

Artificial intelligence (AI), particularly machine learning (ML), shows promise in identifying key influencers and excels in classifying key populations, including pinpointing individuals at higher HIV risk [25-28]. The conventional empirical scales (ES) used a 6-item leadership questionnaire and were previously used to identify GBMSM SHIs and examine their demographic and behavioral characteristics [29]. A limitation of the ES is that there may be bias in the perception of one’s influence or leadership, and it did not consider the social network of these individuals. Thus, more objective and reliable measurements are required [21]. The ML model exhibits better flexibility, whereby it can incorporate more variables and measure SHIs more comprehensively.

To overcome the challenges of the ES to identify SHIs and to tailor this for SD-HIVST, we developed and validated an ML model (ie, an artificial algorithm) to help identify GBMSM SHIs [28]. This trial aimed to compare the effectiveness of SD-HIVST initiated by SHIs identified through the ML model in promoting alters for testing among Chinese GBMSM compared to the ES method. The authors of this paper hypothesized that SHIs identified using the ML model would be more effective in motivating more alters to conduct HIVST in comparison to SHIs identified by the scale.

## Methods

### Ethical Considerations

We registered this trial on the Chinese Clinical Trial Registry website (ChiCTR2000039632). We obtained the institutional review board approval through the Dermatology Hospital of Southern Medical University (2019020[R3]). We obtained different electronic consents from study participants before they started the screening survey, ordered HIVST kits, and uploaded the HIVST results. We provided each participant with 20 RMB (US $3) in compensation through WeChat Pay (China’s most popular social networking app with a mobile payment function) for their time spent completing a survey. We only used participants’ identifiable information for compensation and to eliminate any duplicate entries. We used deidentified data to conduct the analysis.

### Study Setting and Recruitment

We used a quasi-experimental study design. This study was a collaboration between the Social Entrepreneurship to Spur Health team, the Zhuhai Center for Disease Control and Prevention, and the Zhuhai Xutong Voluntary Services Center, a GBMSM community-based organization (CBO).

**Figure 1:**
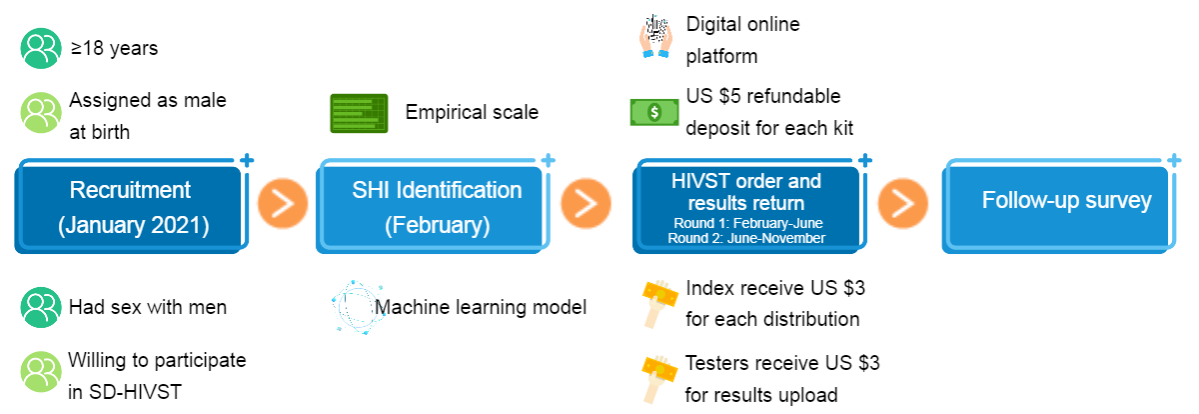
Infographic of the quasi-experimental study: secondary distribution of HIV self-testing (HIVST) kits among gay, bisexual, and other men who have sex with men (GBMSM) in China, from January to November 2021. SHI: sexual health influencer.

The study participants were recruited from 5 provinces in southern China, including Guangdong, Guangxi, Fujian, Hunan, and Jiangxi, by putting recruiting banner ads on BlueD, China’s largest gay social networking app. Interested GBMSM who clicked the banner would answer the presurvey questions to complete the eligibility check. Eligible individuals were then sent an online baseline survey (Figure 1).

### Eligibility Criteria

Participants met the following eligibility criteria: (1) were assigned male sex at birth, (2) were aged 18 years or older, (3) self-reported ever having sex with men, and (4) were willing to engage in the SD-HIVST program.

### Identifying Sexual Health Influencers

To reduce self-report bias, we excluded respondents (survey outliers) who answered with more than 20 sexual partners in the previous 3 months. We randomly assigned eligible individuals to 1 of 2 groups in a 1:1 ratio. Identified SHIs were eligible to engage in the SD-HIVST program. Index participants were SHI who agreed to participate in the study.

A 6-item scale was used to identify SHIs among GBMSM allocated to the ES. This scale was adapted from the one (Cronbach α 0.937) used in our previous study to assess GBMSM’s ability to spread information about sexual health [30,31]. The self-reported scale comprises 6 inquiries designed to evaluate each participant’s involvement in disseminating information about HIV and STIs, both through web-based and offline channels, over the past 3 months. These six questions assess (1) the frequency of their discussions with others regarding HIV and STI, (2) the extent to which they shared information about HIV and STI with others, (3) the number of individuals they informed about HIV and STI, (4) other people’s willingness to seek additional information about HIV and STI from them, (5) whether they or others within their social network were the primary sources of information about HIV and STI, and (6) how often they have sought advice about HIV and STIs. Participants received a total response score ranging from 0 to 24 based on their answers to these questions. Subsequently, SHIs were ranked according to their total scores. More details can be found in the protocol [32].

For the ML model group, we used our developed model to identify SHIs. The model was trained using data from our earlier SD-HIVST program [33]. We used an ensemble approach that involved 4 distinct ML models: logistic regression, random forest, decision tree, and support vector machine. By combining these 4 ML models, we developed our novel ensemble ML model. To ensure the models’ robustness, we applied a 5-fold cross-validation technique and evaluated the average metrics for each model [28]. We gathered data on the selected predictors from the baseline survey and fed it into our already-trained ML model, considering variables selected from social network characteristics, HIV testing, kit application willingness, GBMSM behavior, etc [16].

### Conducting SD-HIVST

Identified SHIs were invited to conduct SD-HIVST on a web-based private platform, which we developed and piloted [15,16]. Index participants accessed the platform by following CBO’s public account on WeChat. CBO posted HIV knowledge and HIVST-related information (eg, kit manufacturer, things to be considered before ordering HIVST kits, and the ordering process) on their WeChat platform. Participants could click the link and read testing-related and HIV-related information. They could also send direct messages to CBO staff based on their needs. In addition, participants could also message research team staff if they had any research-related concerns.

HIVST kits, each including a delivery-tracking code, a result-tracking code, and a user guide, were sent by postal mail to the address provided by the applicant. Testers submitted their results by uploading images to the platform, accessed through a QR code on the package. CBO staff confirmed the results by reviewing the submitted photos. Then, CBO staff would message the verified result to each tester and provide counseling based on the tester’s needs. If a tester had reactive results, the CBO staff would provide information and link them to a confirmation test.

There were 2 rounds of HIVST ordering, and identified SHIs were invited at each round. The first round was from February to June 2021, and the second round was from June to November 2021.

In the first round, index participants could request a maximum of 5 HIVST kits and acquire a unique PR link, allowing them to invite up to 5 alters to order 1 HIVST kit each. In the second round, we lifted the upper limit to 10 HIVST kits and allowed index participants to invite up to 10 alters through the unique PR link. We asked for a refundable deposit of 40 RMB (US $7) per kit at each request.

Index participants would receive 20 RMB (US $3) as incentives for each validated testing result of alters. Furthermore, all unique testers earned 20 RMB (US $3) after verifying their testing results.

### Outcomes

We identified unique alters as individuals who received tests from index participants and reported results using unique cell phone numbers. If alters were tested twice, we kept their most up-to-date results. We further classified alters who self-reported having never tested for HIV before this research as unique newly tested alters.

Primary outcomes included (1) the number of unique alters motivated by index participants in each group and (2) the number of unique newly tested alters motivated by index participants in each group. They were assessed when HIVST testers completed the return survey. Secondary outcomes included (1) the number of testers tested with HIV-reactive results, (2) the number of unique alters motivated by index participants categorized in subgroups, and (3) the economic cost comparison between the ES and the ML model.

### Sample Size Calculation

We calculated a sample size of 400 SHIs with 80% power, a 2-sided α=.05, and a 20% loss to follow-up. According to our preliminary study data, we assumed the mean difference (MD) was 1 between the 2 groups in unique alters motivation and 0.3 in newly tested alters motivation [32]. We conducted sample size estimation using PASS software (version 16.0; NCSS, LLC).

### Missing Data and Sensitivity Analysis

No index participants dropped out of the study during the entire study period. In data analysis, we only included baseline survey data related to index participants’ sociodemographics without using follow-up survey data, and the baseline data was complete without any missing data. When uploading test results, some alters might choose their role as “index participants” to skip answering the alters’ survey through which we collected whether they were newly tested. Since CBO staff worked closely with index participants, they verified the identity of each uploaded result and made a note in the database for reference researchers.

We conducted sensitivity analyses under two assumptions: (1) considering all individuals who skipped the survey as newly tested, and (2) considering all individuals who skipped the survey as not newly tested.

### Statistical Methods

We used the Shapiro-Wilk method to test the data distribution of baseline characteristics of index participants and presented the mean (SD) for the primary outcomes of each group. We calculated MD between groups and used bootstrapping to estimate corresponding 95% CIs. We used negative binomial regression for primary outcome analyses and adjusted covariates, including age group, income, education, residence permit, marital status, sexual orientation, and residence. We performed the same regressions on subgroup analysis, whereby we classified index participants based on their age, sexual orientation, sexual orientation disclosure, residence permit, education, and province of residence. We believe negative binomial regression is more suitable than other methods for the following reasons: (1) the variance of the count number (ie, outcomes) is larger than the mean value (Table 1), thus assumptions of Poisson regression are not met; (2) we conducted tests for the presence of excess 0s in the data. The results indicated that there were no excess 0s, suggesting that the data was not a suitable fit for 0-inflated negative binomial regression.

We considered *P*<.05 as statistically significant and performed data analysis in RStudio (version 1.2.5033; Posit).

We used a microcosting method to assess the economic cost (ie, the cost of all resources needed to implement the testing models) from the perspective of a health provider. We kept track of the resources used throughout the experiment and classified cost items. We collected cost data (Table S1 in Multimedia Appendix 1) and analyzed costs. All expenses are expressed in 2021 US dollars using OANDA currency conversions (US $1=6.51 RMB). We conducted the cost analysis in Excel 2019 (Microsoft Corporation).

## Results

### Overview

We received 2150 recruitment surveys from January 7 to January 28, 2021. After excluding 5% outliers from 1922 respondents, we identified 1828 unique participants with verified contact information. We randomly assigned 1828 participants to each group (ML model=914 and ES=914). We further identified 465 SHIs in the ES and 465 SHIs in the ML model group.

**Figure 2:**
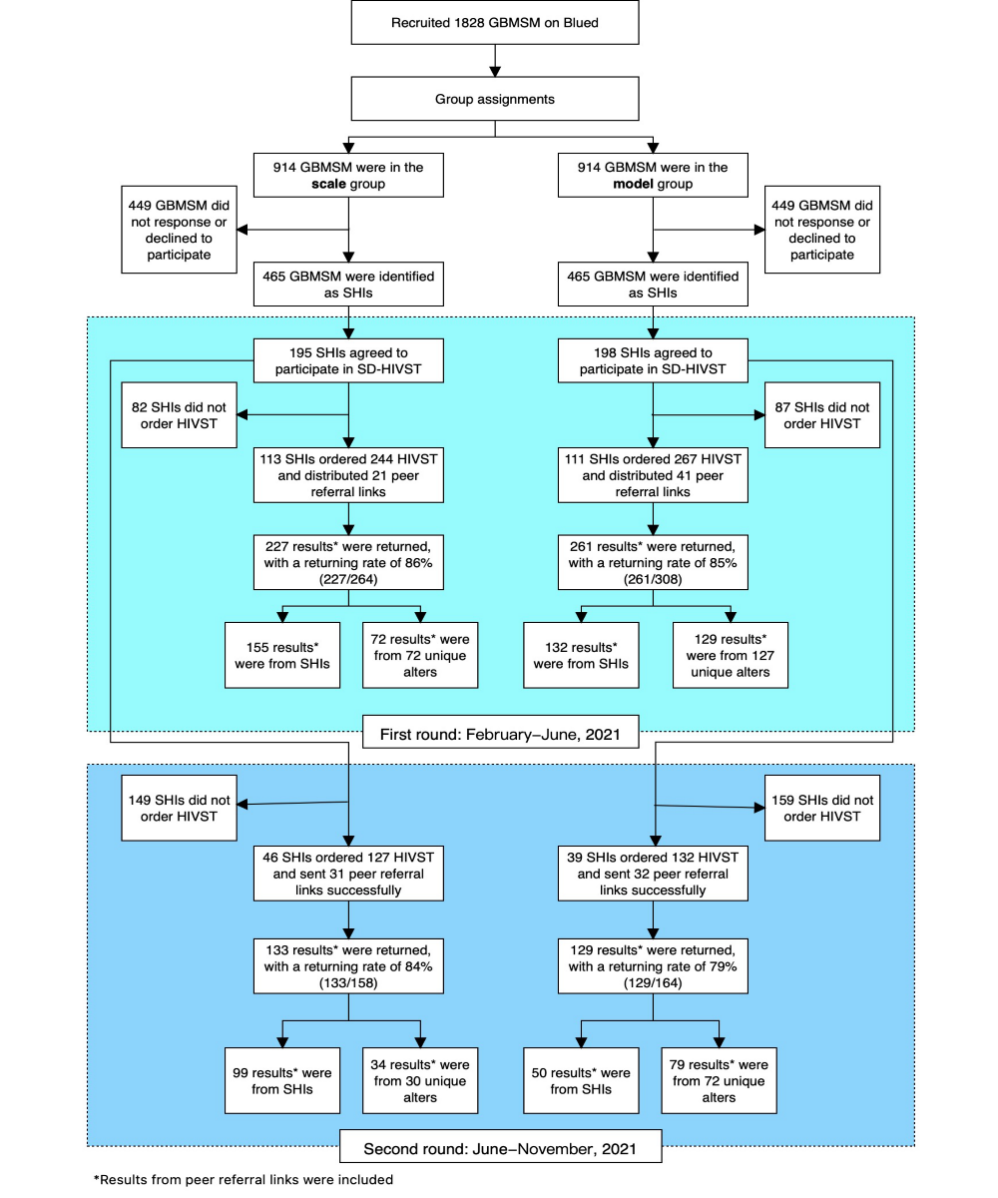
Flowchart of the quasi-experimental study: secondary distribution of HIV self-testing kits among gay, bisexual, and other men who have sex with men (GBMSM) in China, from January to November 2021. SHI: sexual health influencer.

After being invited to participate in SD-HIVST, 393 identified SHIs (ES=195 and ML model=198) further confirmed their interests, and 229 index participants (ES=116 and ML model=113) initiated SD-HIVST and were included in this study. SD-HIVST was implemented from February 2021 to November 2021 (Figure 2).

### Sociodemographic Characteristics

In total, 229 index participants ordered SD-HIVST, with 59.5% (116/195) in the ES and 57.1% (113/198) in the ML model ordering HIVST kits on the web.

The social demographic characteristics were similar between the 2 groups, except for education status (Table 1). Overall, the mean age of index participants was 27.58 (SD 7.75) years, 76% (175/229) of them obtained at least a college degree, 87.3% (200/229) were single, 72.9% (167/229) self-identified as gay, 68.6% (157/229) disclosed their sexual orientation to people who are not gay, and 91.7% (210/229) ever tested for HIV.

**Table 1:** Baseline characteristics of index participants (n=229) in China, stratified by the empirical scale (ES) group versus the machine learning (ML) model group, from February to November 2021. A 2-tailed t test was applied to compare ages between the ES and ML models. Chi-square tests were applied to test differences in categorical characteristics between the ES and ML models.

Characteristics			ES (n=116)^a^		ML model (n=113)^b^		Overall (N=229)		*P* value	
Age (years), mean (SD)			27.43 (7.91)		27.73 (7.61)		27.58 (7.75)		.77	
**Age (years), n (%)**										
	<30	81 (69.8)		80 (70.8)		161 (70.3)				
	≥30	35 (30.2)		33 (29.2)		68 (29.7)				
**Education, n (%)**										0.047
	High school or below	34 (29.3)		20 (17.7)		54 (23.6)				
	College	73 (62.9)		88 (77.9)		161 (70.3)				
	Master’s degree or above	9 (7.8)		5 (4.4)		14 (6.1)				
**Annual income (US $), n (%)**										.68
	<2700	19 (16.4)		13 (11.5)		32 (14.0)				
	2700-5400	19 (16.4)		17 (15.0)		36 (15.7)				
	5401-9000	30 (25.9)		26 (23.0)		56 (24.5)				
	9001-14,400	22 (19.0)		27 (23.9)		49 (21.4)				
	>14,400	26 (22.4)		30 (26.5)		56 (24.5)				
**Sexual orientation, n (%)**										.83
	Gay	84 (72.4)		83 (73.4)		167 (72.9)				
	Bisexual	26 (22.4)		26 (23.0)		52 (22.7)				
	Unsure	6 (5.2)		4 (3.5)		10 (4.4)				
**Sexual orientation disclosure^c^** ^c^										.40
	No	40 (34.5)		32 (28.3)		72 (31.4)				
	Yes	76 (65.5)		81 (71.6)		157 (68.6)				
**Marital status^d^, n (%)**										.16
	Engaged or married	12 (10.3)		8 (7.1)		20 (8.7)				
	Single	97 (83.6)		103 (91.2)		200 (87.3)				
	Separated, divorced, or widowed	7 (6.0)		2 (1.8)		9 (3.9)				
**Residence, n (%)**										.40
	Guangdong Province	68 (58.6)		59 (52.2)		127 (55.5)				
	Other provinces	48 (41.4)		54 (47.8)		102 (44.5)				
**Residence permit, n (%)**										.10
	Rural	72 (62.1)		57 (50.4)		129 (56.3)				
	Urban	44 (37.9)		56 (49.6)		100 (43.7)				
**Condomless sex with other men in the past 3 months^e^, n (%)**										.87
	Yes	47 (40.5)		48 (42.5)		95 (41.5)				
	No	69 (59.5)		65 (57.5)		134 (58.5)				
**Ever tested for HIV, n (%)**										.17
	Yes	103 (88.8)		107 (94.7)		210 (91.7)				
	No	13 (11.2)		6 (5.3)		19 (8.3)				

^a^A total of 59.5% (116/195) participants in the ES ordered HIV self-testing kits on the web.

^b^A total of 57.1% (113/198) participants in the ML model ordered HIV self-testing kits on the web.

^c^Index participants disclosed their sexual orientation to other people who were self-identified as not gay.

^d^Marital status refers to heterosexual marriage.

^e^In the past 3 months refers to October 2020 to January 2021.

### Application (First Round)

Overall, 224 index participants ordered HIVST kits through the platform. In the ES, 113 index participants ordered 244 kits and referred to 21 PR links. We received 227 results, with a return rate of 85.7% (227/265), among which 72 results were from 72 unique alters.

In the ML model, 111 index participants ordered 267 kits and referred 41 PR links to alters. We received 261 results, with a return rate of 84.7% (261/308), among which 129 results were from 127 unique alters.

A total of 84.1% (95/113) index participants in the ES completed the 3-month follow-up survey, while 86.5% (96/111) index participants in the ML model completed the survey.

### Application (Second Round)

In the second round, 85 index participants ordered kits through the platform. A total of 46 index participants in the ES ordered 127 kits and referred to 31 PR links. We received 133 results, with a return rate of 84.2% (133/158), among which 34 results were from 30 unique alters.

In the ML model, 39 index participants ordered 132 kits and referred 32 PR links to alters. We received 129 results, with a return rate of 78.6% (129/164), among which 79 results were from 72 unique alters.

A total of 87% (40/46) index participants in the ES and 87% (34/39) participants in the ML model completed the 3-month follow-up survey.

### Unique Alter Motivation Results

Table 2 presents the primary outcome results for 229 index participants in the 6-month study period on the effect of motivating unique alters and newly tested alters. We estimated a total effect from 2 rounds, with separate effects for the first and second rounds.

Overall, index participants in the ML model motivated an average of 1.76 (199/113, SD 3.97) unique alters to conduct HIVST compared with 0.88 (102/116, SD 1.84) in the ES, with an MD of 0.88 (95% CI 0.02-2.22). Index participants in the ML model were more likely to motivate unique alters than those in the ES (adjusted incidence rate ratio [aIRR] 1.77, 95% CI 1.07-2.95).

**Table 2:** The effectiveness in motivating alters and newly tested alters to conduct HIV self-testing (HIVST), comparing index participants in the empirical scale (ES) group to those in the machine learning (ML) model group in China, February to November 2021. We kept the unique index participants when analyzing the outcomes of the first and second rounds. Therefore, the number of overall index participants is not equal to the sum of the absolute number of 2 rounds of index participants.

Outcomes and number of index participants			Number of unique alters motivated by each index participant		Number of newly tested alters motivated by each index participant
**First round and second round**					
	ES (n=116), n	102		44	
	ES (n=116), mean (SD)	0.88 (1.84)		0.38 (0.89)	
	ML model (n=113), n	199		82	
	ML model (n=113), mean (SD)	1.76 (3.97)		0.73 (2.32)	
	ES (reference) versus ML model, MD^a^ (95% CI)	0.88 (0.02 to 2.22)		0.35 (–0.17 to 0.99)	
	ES (reference) versus ML model, aIRR^b^ (95% CI)	1.77 (1.07 to 2.95)^c^		1.49 (0.74 to 3.02)	
**First round (from February to June 2021)**					
	ES (n=113), n	72		32	
	ES (n=113), mean (SD)	0.64 (1.39)		0.28 (0.70)	
	ML model (n=111), n	127		41	
	ML model (n=111), mean (SD)	1.14 (1.80)		0.37 (0.77)	
	ES (reference) versus ML model, MD (95% CI)	0.50 (–0.01 to 1.02)		0.09 (–0.17 to 0.34)	
	ES (reference) versus ML model, aIRR (95% CI)	1.85 (1.10 to 3.12)^c^		1.56 (0.75 to 3.23)	
**Second round (from June to November 2021)**					
	ES (n=46), n	30		12	
	ES (n=46), mean (SD)	0.65 (1.48)		0.26 (0.74)	
	ML model (n=39)	72		41	
	ML model (n=39), mean (SD)	1.85 (3.98)		1.05 (3.03)	
	ES (reference) versus ML model, MD (95% CI)	1.20 (–0.25 to 3.20)		0.79 (–0.17 to 2.17)	
	ES (reference) versus ML model, aIRR (95% CI)	1.75 (0.83 to 3.69)		—^d^	

^a^MD: mean difference.

^b^aIRR: adjusted incidence rate ratio. A negative binomial model was used to calculate the incidence rate ratio (IRR). aIRR was adjusted for age (<30 and ≥30 years), income, education, marital status (referring to heterosexual marriage), sexual orientation, and residence.

^c^*P*<.05.

^d^The Hauck-Donner effect occurred, causing the estimated coefficients to be in extreme situations.

### Newly Tested Alters Motivation Results

Throughout 2 rounds, index participants in the ML model averagely motivated 0.73 (82/113) unique newly tested alters, compared with 0.38 (44/116) in the ES. The performance of index participants in the ML model was slightly better than that in the ES (MD 0.35, 95% CI –0.17 to 0.99), but the difference between these 2 groups was not statistically significant. (Table 2.) We identified 4 alters who chose their role as index participants. Our sensitivity analysis results showed a similar pattern.

### Subgroup Analysis Results

Table 3 reveals subgroup analysis results. Index participants in the ML model who were aged 30 years or younger, not gay, or residents of Guangdong were more likely to motivate unique alters to conduct HIVST than those who were in the ES.

**Table 3:** The effectiveness in motivating alters to conduct HIV self-testing (HIVST), comparing index participants in the empirical scale (ES) group to those in the machine learning (ML) model group, stratified by their baseline demographics in China, from February to November 2021.

Variables			ES (reference) versus ML model, aIRR^a^ (95% CI)
**Age (years)**			
	<30	2.06 (1.09-3.91)^b^	
	≥30	—^c^	
**Sexual orientation**			
	Gay	1.51 (0.85-2.69)	
	Others^d^	4.16 (1.31-14.60)^b^	
**Sexual orientation disclosure** ^e^			
	No	2.20 (0.82-6.06)	
	Yes	1.50 (0.80-2.83)	
**Residence permit**			
	Rural	1.83 (0.98-3.44)	
	Urban	1.37 (0.65-2.95)	
**Education**			
	High school or below	2.12 (0.94-4.87)	
	College or above	1.69 (0.92-3.09)	
**Province**			
	Guangdong Province	1.81 (0.97-3.42)^b^	
	Outside Guangdong	1.74 (0.76-3.97)	

^a^aIRR: adjusted incidence rate ratio. A negative binomial model was used to calculate the incidence rate ratio (IRR). aIRRs were adjusted for age, income, education, marital status, sexual orientation, and residence.

^b^*P*<.05.

^c^The Hauck-Donner effect occurred, causing the estimated coefficients to be in an extreme situation.

^d^Others include bisexual and unsure.

^e^Index participants disclosed their sexual orientation to other people who were self-identified as not gay.

### HIVST Reactive Results

Among 14 testers who were newly tested HIV reactive, 6 were index participants (3 in the ES and 3 in the ML model), and 8 were alters (4 in the ES and 4 in the ML model). CBO staff followed up with all testers with reactive results.

### Cost Analysis

The total economic cost of implementing the SD-HIVST was US $6547.12 for the ES and US $6767.19 for the ML model. When comparing the ML model and ES approaches, the ML model is more effective in encouraging participants to get tested for HIV, but it is not cost-saving. The incremental cost to gain an additional tested participant is US $2.27, and for a newly tested participant, the incremental cost is US $5.79 (Table 4).

**Table 4:** Cost analysis evaluating the effectiveness in motivating alters and newly tested alters to conduct HIV self-testing (HIVST), comparing index participants in the empirical scale (ES) group to those in the machine learning (ML) model group, from February to November 2021.

Analysis	ES	ML model
Cost per alters (US $)	64.19	37.05
Incremental cost (US $)	Reference group	–27.13
Unique alters motivated by each index participant, n	102	199
ICER^a^	N/A^b^	Dominated^c^
Total economic cost (US $)	6547.2	6767.19
Incremental cost (US $)	N/A	220.07
Newly tested alters motivated by each index participant being tested, n	44	82
ICER	N/A	5.79

^a^N/A: not applicable.

^b^ICER: incremental cost-effectiveness ratio.

^c^Dominated means sexual health influencers in the ML model are more effective and cost-saving compared to their counterparts in the ES model.

## Discussion

### Overview

We conducted a quasi-experimental study among Chinese MSM and found that, overall, SHIs identified by the ML model can influence more individuals through their social network to conduct HIVST than SHIs identified by the conventional scale, highlighting the potential of artificial AI to enhance the efficiency of testing approaches. Building on the evidence that monetary incentives and peer referral can amplify the effect of SD-HIVST, this study extends the literature by comparing the effectiveness of the ES and a ML model in identifying SHIs for secondary distribution and reporting results on the total number of unique alters and newly tested alters motivated by the identified SHIs [15,16]. In addition, we acknowledged the important role that community engagement plays in promoting HIV testing among GBMSM. Identifying SHIs to implement community-led interventions could expand HIV testing services. As influential community members, SHIs build on their potential to support their peers in improving health awareness and behaviors, thereby engaging more individuals from their social networks and facilitating HIVST uptake.

### Principal Findings

The ML model was more effective than the ES in engaging unique alters for HIVST during SD-HIVST. This is aligned with the results of a previous study using an interactive deep learning approach to identify HIV influencers; that is, not only ML models can detect HIV-related digital social influencers, but also ML models show higher accuracy in identification [34]. Despite studies showing that SHIs and noninfluencers can promote HIV testing within their social networks, the groups they can reach differ, particularly alters of SHIs who sought out more casual sexual partners, which increased their risk of HIV infection [22,35]. In light of this, it is promising to integrate them into the current SD-HIVST programs to facilitate HIV testing uptake and case identification further. Both the ML model and the ES revealed modest differences in the motivation of newly tested alters. The results of the ML model were statistically insignificant in promoting first-time testing compared to the ES; this may be due to the power of the study. However, in terms of implementation, SHIs identified by the ML model motivated a larger number of newly tested alters and promoted first-time testing coverage. Previous studies have demonstrated that first-time testing among GBMSM is associated with sociodemographic and other factors such as the number of sexual partners, condomless sex, and STI infection history [30,31]. Further refinement of the interventions may be warranted to reach more newly tested alters.

### Comparison to Previous Work

Evidence-based HIV prevention strategies can be significantly enhanced through interdisciplinary collaboration between communities and academia [33]. At the community level, our research was rooted in strong partnerships between the academic and community sectors. We closely worked with the local gay-led CBO to evaluate the interventions designed for specific populations, ensuring a collaborative and informed approach to this study. A review paper demonstrated that AI, exemplified by Van Heerden et al [36] mobile app using natural language processing and Brown et al [37] partner notification model with unconditional logistic regression, shows promise in enhancing HIV counseling, testing, and partner notification strategies [33,36,37]. Our research extends the existing literature on the application of AI in HIV testing, specifically concentrating on identifying SHIs and expanding the effectiveness of the current SD-HIVST program.

Additionally, several studies have been done on the feasibility and acceptability of digital interventions, and further strategies to accelerate digital interventions for HIV prevention are needed [38,39]. ML as a data science approach has sparked significant research interest in developing digital health interventions [33,40]. The strength of our ML model lies in both accuracy and flexibility. In building the ML model, more specific variables were incorporated and weighted differently to define SHIs, such as the number of copies of HIVST wanted, the intention to deliver HIVST, and the willingness to obtain PR links [41]. Further, the absence of the data sets from the previous study for the ES or social network-based simulation optimization models renders them less accurate than the ML model in identifying SHIs during SD-HIVST [42].

### Strengths and Limitations

This study also has several limitations. First, this study is quasi-experimental, which may be subject to selection bias. Only half of the identified SHIs agreed to participate, and only one-quarter initiated the HIV self-test kit application process. However, the social demographic characteristics were comparable between the 2 study arms. Second, the ML model did not outperform the ES in promoting newly tested alters. The main reason for this could be the selection of parameters when building the ML model that primarily motivates unique alters. Future research regarding motivating the newly tested alters requires additional consideration of the selection of variables and the weight given to them during modeling. Finally, compared to our previous studies, this study reported a relatively lower result return rate among the testers, mainly due to a much lower deposit, which reduces the “sunk cost” for the testers so that they are less inclined to return the results [15,16]. A higher deposit fee may be advisable for similar programs to ensure a higher rate of returns. However, our sensitivity analysis indicated that the impact of these nonreturns has a minimum impact on this study results.

### Future Directions

As for implications, this study demonstrated that interventions led and delivered by CBO could facilitate participation. The local GBMSM-friendly CBO undertook the health service delivery in this study, including the construction and maintenance of the HIVST kit ordering platform, HIVST kit distribution, counseling, results interpretation, and linkage to care services. This was consistent with a systematic review that community engagement interventions are beneficial for marginalized populations in increasing health behaviors, health outcomes, participant self-efficacy, and perceived social support [43]. Particularly in COVID-19, our innovative digital health intervention can guarantee access to health services while saving costs. This ML model method of identifying SHIs may also apply to other regions and other interventions, as it has good extensibility and flexibility. Depending on the interventions (including but not limited to SD-HIVST), we can build the ML model based on different selected associated variables.

### Conclusion

Identifying SHIs can enhance the effectiveness of social network–based intervention among GBMSM. We found that SHIs identified by the ML model can motivate more individuals to conduct HIVST than those identified by the ES in SD-HIVST programs among Chinese GBMSM. This study innovatively adopted data science techniques to develop the ML model for identifying SHIs and delivering interventions in collaboration with community-based organizations. This strategy can be adapted and implemented to improve HIV care services.

## Appendix

Multimedia Appendix 1 Table S1 and other supplementary material.

## Abbreviations

AI: Artificial intelligence
aIRR: adjusted incidence risk ratio
CBO: community-based organization
ES: empirical scales
GBMSM: gay, bisexual, and other men who have sex with men
HIVST: HIV self-testing
MD: mean difference
ML: machine learning
MSM: men who have sex with men
PR: peer referral
SD-HIVST: secondary distribution of HIV self-testing
SHI: sexual health influencer
STI: sexually transmitted infection
UNAIDS: Joint United Nations Programme on HIV/AIDS
WHO: World Health Organization

## Data Availability

The data sets generated or analyzed during this study are not publicly available due to identifiable information related to participants but are available from the corresponding author upon reasonable request.
