# Supplementary material for: A Machine Learning Model for Identifying Sexual Health Influencers to Promote the Secondary Distribution of HIV Self-Testing Among Gay, Bisexual, and Other Men Who Have Sex With Men in China: Quasi-Experimental Study
Source: JMIR Public Health Surveill. 2024 Apr 24;10:e50656. doi: 10.2196/50656 (PMC11079758; doi:10.2196/50656)
Supplement: Multimedia Appendix 1 [file publichealth_v10i1e50656_app1.docx]

**Supplement materials**

Supplement Table S1: Cost items

Protocol Amendments

**Supplement Table S1: Cost items (in 2021 USD)**

|  |  | **ES** |  | **MLM** |  |
| --- | --- | --- | --- | --- | --- |
| **Startup Cost** | **Unit cost** | **Number** | **Total cost** | **Number** | **Total cost** |
| *Trainer* |  |  |  |  |  |
| SESH research assistant A | 6.06 | 2 | 12.13 | 2 | 12.13 |
| SESH research assistant B | 6.06 | 2 | 12.13 | 2 | 12.13 |
| *Participants* |  |  |  |  |  |
| Part-time worker A | 0.10 | 2 | 0.19 | 2 | 0.19 |
| Part-time worker B | 0.13 | 2 | 0.26 | 2 | 0.26 |
| *Total startup cost* |  |  | 24.70 |  | 24.70 |
| **Variable cost** |  |  |  |  |  |
| *Broad Labor Activities (HIVST kits)* |  |  |  |  |  |
| Assembly of self-testing kit packages per package | 0.26 | 422 | 107.96 | 472 | 120.75 |
| Self-testing kit delivery per kit | 0.17 | 422 | 73.70 | 472 | 82.43 |
| Provide project counselling for indexes per weekday | 7.62 | 99 | 753.98 | 99 | 753.98 |
| Review index details per weekday | 3.96 | 99 | 392.06 | 99 | 392.06 |
| Contact index for data correction (e.g, age) per weekday | 3.96 | 99 | 392.06 | 99 | 392.06 |
| Self-testing result interpretation and return per record | 0.38 | 361 | 138.81 | 389 | 149.58 |
| Information check and deposit refund per weekday | 3.96 | 99 | 392.06 | 99 | 392.06 |
| HIV/Syphilis positive result notification and follow-up per positive tester | 2.83 | 16 | 45.26 | 23 | 65.06 |
| Contact testers regarding invalid results/kits use incorrectly per tester with invalid result | 0.44 | 18 | 7.83 | 23 | 10.01 |
| Follow up with testers who re-submit test results per tester with invalid result | 0.47 | 18 | 8.49 | 23 | 10.84 |
| 3-month follow-up survey per weekday | 7.62 | 99 | 0.00 | 99 | 0.00 |
| Review qualified testers receiving monetary incentives per tester | 0.11 | 227 | 25.04 | 261 | 28.79 |
| Self-testing publicity information compile and release per ad | 7.66 | 1 | 7.66 | 1 | 7.66 |
| Attend/ Prepare HIVST team meeting per weekday | 6.43 | 99 | 636.98 | 99 | 636.98 |
| Monitor/supervise intervention per weekday | 6.43 | 99 | 636.98 | 99 | 636.98 |
| Database maintenance per weekday | 12.87 | 99 | 1273.97 | 99 | 1273.97 |
| *Broad Labor Activities (PR links)* |  |  |  |  |  |
| Review referred alters application details per referred alter | 0.09 | 52 | 4.90 | 73 | 6.88 |
| Generate personal QR code per month | 3.83 | 9 | 34.47 | 9 | 34.47 |
| Contact index for QR code download per index | 0.09 | 115 | 10.84 | 124 | 11.69 |
| Record and organize referred alters information per referred alter | 0.09 | 52 | 4.90 | 73 | 6.88 |
| *Broad Non-labor Activities* |  |  |  |  |  |
| Total PR self-testing kit cost | 4.03 | 52 | 209.59 | 73 | 294.23 |
| Okamoto condom (2 count) | 0.61 | 57 | 34.93 | 57 | 34.93 |
| Lubricant oil (50ml) | 0.39 | 111 | 43.34 | 111 | 43.34 |
| Express fee | 2.20 | 232 | 509.30 | 264 | 579.55 |
| Pen | 0.77 | 1 | 0.77 | 1 | 0.77 |
| *Total variable cost* |  |  | 5745.89 |  | 5965.96 |
|  |  |  |  |  |  |
| **Fixed Cost** |  |  |  |  |  |
| Working Buildings | 112.73 | 1 | 112.73 | 1 | 112.73 |
| Work and management expenses | 136.43 | 1 | 136.43 | 1 | 136.43 |
| Office Equipment | 119.87 | 1 | 119.87 | 1 | 119.87 |
| Capacity Building | 407.50 | 1 | 407.50 | 1 | 407.50 |
| *Total fixed cost* |  |  | 776.53 |  | 776.53 |
| **Total economic cost** |  |  | 6547.12 |  | 6767.19 |

ES = Empirical scale group. MLM = Machine learning model group. PR = Peer referral. USD = United States dollar.

**Protocol Amendments**

Protocol title: Effectiveness of sexual health influencers identified by an ensemble machine learning model in promoting secondary

distribution of HIV self-testing among men who have sex with men in China: study protocol for a quasi-experimental trial

Revisions to V1.0: 12 Jun 2021 (Original protocol)

Date: V2.0: 6 Jan 2023 (Amendment 01)

| Change | Rationale | Affected Protocol Sections |
| --- | --- | --- |
| We revised our first secondary outcome from “the mean number of tested alters motivated by each index in each group with HIV reactive results” to “the number of testers tested with HIV-reactive results and syphilis results” | Due to a small sample size, we chose to report the numbers of testers with HIV-reactive results. | Outcome  <Page 6> |
| We added a secondary outcome “3) economic cost comparison between the ES and the MLM.” | We wanted to further explore the differences of economic cost between two groups. A cost evaluation could provide cost-effectiveness evidence for decision-making as well as the MLM implementation in the future in order to allocate limited healthcare resources more effectively. | Outcome  <Page 6> |
